# Editorial: The Mental Capacity Act 2005—Ten Years On

**DOI:** 10.1093/medlaw/fww032

**Published:** 2016-12-22

**Authors:** Amel Alghrani, Paula Case, John Fanning

**Affiliations:** Department of Law, University of Liverpool, Liverpool, UK

More than a decade has passed since the Mental Capacity Act (MCA) received royal assent. Described as a ‘visionary piece of legislation’, the MCA was a significant landmark on the legal landscape.^1^ It represented a triumph of autonomy by recognising that, as far as possible, people should play an active role in decisions about their welfare. At the core of the MCA is the fundamental principle that a person must be assumed to have decision-making capacity unless it is established that he lacks it.^2^ The law, therefore, assumes that *everyone* has the ability to act and take decisions in accordance with their own interests, and affords primacy to individual priorities over paternalistic imperatives. Where a person (‘P’) lacks capacity—whether for reasons of learning disability, dementia, brain injury, or some other impairment of or disturbance in the functioning of the mind or brain—the MCA permits decision-makers to act on behalf of P in accordance with his ‘best interests’.^3^ This means that, among other things, decision-makers must take into account P’s past and present wishes and feelings, his beliefs and values, and any other factors that P would be likely to consider, in order to act in a way which would likely give expression to P’s autonomy.^4^ In this way, the MCA seeks to empower people to make decisions for themselves, protect the vulnerable from the excesses of paternalism, and engineer a cultural shift in attitudes to mental impairment and incapacity.

The scope of the MCA’s application was certainly ambitious. A major challenge for its drafters was to devise a coherent framework through which decision-makers could determine whether P lacks capacity to make her own decisions and what to do when she is found to lack that capacity. Perhaps unsurprisingly, the MCA’s stipulations apply in a wide range of circumstances, pertaining to both lay and professional decision-makers (ie regardless of whether the decision-maker is a family member, healthcare professional, social worker, care worker, lawyer, or judge) and concerning a diverse array of situational contexts (eg from relatively trivial, to life-changing—and even life-ending—decisions). With this in mind, few would dispute that the MCA is the defining statute of English medical law in the twenty-first century.

However, as its 10-year anniversary approached, the MCA appeared to have a mixed record. On one hand, in *Aintree University Hospitals NHS Foundation Trust v James,*^5^ the Supreme Court was widely acclaimed for insisting that the perspective of incapacitated patients should lie at the heart of the MCA’s ‘best interests’ test. *James,* therefore, demonstrated explicit judicial acceptance of the fundamental principle that decisions should be taken through the prism of patients’ interests rather than for reasons of practical expediency or according to paternalistic instincts. On the other hand, the House of Lords Select Committee for Health embarked on a wide-ranging review of the Act’s implementation and observed that its ‘empowering ethos’ had not been delivered:

Our evidence suggests that capacity is not always assumed when it should be [and] … . The concept of unwise decision-making faces institutional obstruction due to prevailing cultures of risk-aversion and paternalism.^6^

It occurred to the editors of this special issue of the *Medical Law Review* that the tenth anniversary of the introduction of the MCA was an opportune moment to reflect on the 2005 Act’s record so far and to evaluate its implications for the future. To what extent has the MCA changed medical practice in England and Wales since its introduction? How effectively has it been implemented? To what extent has the MCA’s unashamed emphasis on autonomy transformed professional culture? This special issue brings together a range of papers from an array of academic and practitioner authors which reflect on the MCA’s first decade at large. Many of the papers focus on judgments from the Court of Protection (CoP), a ‘new’ court created by section 45 of the MCA, with jurisdiction to deal with decisions for anyone assessed as lacking decision-making capacity. Judgments concerning the provisions of the MCA were initially slow to emerge, and the CoP came in for criticism from the press, accused of being a dark, secret court where life-changing decisions were made away from public scrutiny.^7^ Transparency reforms to the CoP in 2014 have meant that the paucity of reported cases has, to an extent, been remedied,^8^ although increased transparency has raised fresh concerns regarding intense media interest in some of the reported cases and the impact on family members at an already difficult time.^9^

In excess of 90% of applications made to the CoP concern non-contentious property and financial decisions.^10^ Most of the remaining applications concern whether ‘health and welfare’ decisions need to be made on behalf of P and, if so, what form they should take. The ‘health and welfare’ jurisdiction of the CoP is made up of emotionally charged and disturbing cases concerning individuals, often failed by their bodies and minds, and sometimes by those responsible for caring for them. Increasingly, judgments report the conversations and correspondence between the judge and ‘P’, the person at the centre of these proceedings. P’s participation in CoP proceedings can provide an important safeguard against excessive ‘medicalism’ or paternalistic professional practices. But it is often the case that P will lack litigation capacity and is heavily dependent on their ‘litigation friend’ to afford them a voice. Neil Allen, Peter Bartlett, and Alex Ruck Keene in ‘Litigation Friends or Foes? Representation of “P” Before the Court of Protection’ provide a detailed interrogation of the role of ‘litigation friends’. Their article highlights the distinction between ‘representing P’ (putting P’s views before the court) and ‘acting on P’s behalf’, to highlight the conflicts within this role which can prevent P’s wishes and feelings being fully represented in court. The authors argue that P ought to have the same right as anyone else to advance ‘unwise’ arguments, subject only to the case management powers of the court, whereas practice has developed according to an assumption that the litigation friend will adopt a ‘best interests’ approach to the arguments to be advanced ‘on behalf of P’. This article is informed by many years of academic and expertise and practitioner experience of the workings of CoP proceedings and provides some fascinating observations on the history and legal principles pertaining to the role of the litigation friend, leaving readers to consider the fit of current practice with the Supreme Court judgment in *James.*

Many of the MCA’s provisions attempted to capture the pre-existing common law principles on how a person’s mental capacity was to be assessed, and included a number of provisions which collectively guard against decision-making capacity being determined by the outcome of the decision.^11^ In ‘Dangerous Liaisons: Psychiatry, Language and Law in the Court of Protection’, Paula Case engages in an analysis of the relevance of clinical judgement to the assessment of capacity under the MCA provisions. Case argues that heavy reliance on psychiatry has resulted in clinical language being imported into the business of capacity assessment, specifically the language of ‘insight’, a term with a contested history, which is not mentioned in the Act or in the accompanying Code of Practice. There is a risk that the application of the statutory criteria for incapacity is sometimes obscured by the non-statutory, clinical terminology of ‘insight’ used by expert witnesses in CoP proceedings. In Case’s view, its use in evidence on capacity is often not clearly mapped onto the statutory criteria and it has the potential to cloak value judgments regarding P’s non-cooperative and obstructive behaviour in the apparel of ‘clinical fact’. Uncritically accepting the use of ‘insight’ in expert evidence on capacity assessment without clear attempts to map this lack of insight onto the statutory test for incapacity is argued to potentially give credence to therapeutic values which seem in conflict with many of the autonomy promoting provisions of the MCA. While this article is not the first to draw attention to the use of the nebulous term ‘insight’ as a proxy for capacity, Case offers an analysis of its use in post-MCA judgments.

It is understandable that much scrutiny of the Act has congregated on the theme of the ‘best interests’ test and the extent to which the emergent jurisprudence has attached weight to P’s wishes and feelings. Not long after the MCA came into being, commentators began to question whether the MCA had already exceeded its shelf life. In 2006, the United Nations was agreeing the final text of the Convention on the Rights of Persons with Disabilities (UNCRPD) which came into effect in 2008. The Convention is based on a social model of disability and promises access to equality, dignity, and inclusion for those with disabilities. A number of its provisions indirectly challenge the orthodoxy of the MCA (an issue which also features in Allen, Bartlett, and Ruck Keene’s paper) and soon, serious questions were being asked about the compatibility of the MCA with UNCRPD values and requirements. The House of Lords Select Committee on the MCA chose not to become ensnared in debates about the MCA’s compatibility, but preferred to conclude that better alignment with the UNCRPD represented a ‘reasonable aim’.^12^ The most contentious provision so far appears to be Article 12 of the UNCRPD which requires that those with disabilities should enjoy legal capacity on an equal basis with others. Paul Skowron in ‘The Implications of Meno’s Paradox for the Mental Capacity Act 2005' explores when, if ever, it is right to make a decision on behalf of another person against their will. He shares concerns with many of the other authors in this special issue regarding the tensions which exist between the MCA framework (which appears to endorse decisions made against P’s will and preferences with the proviso that they are ‘objectively’ in P’s best interests) and the UNCRPD (read by many as requiring a framework for decision-making which is more closely aligned to P’s will and preferences^13^). Mary Donnelly’s paper, ‘Best Interests in the Mental Capacity Act: Time to Say Goodbye?’ explores some of these tensions further. Donnelly critiques the ‘will and preferences’ paradigm for decision-making proposed by The Committee on the Rights of Persons with Disabilities in General Comment No.1, but rather than dismissing the best interests test as ‘non-compliant’ with the UNCRPD, she offers a qualified defence of ‘best interests’, advocating for a stronger statement of the primacy of P’s wishes and feelings in the Act.^14^ She observes that although post-MCA judgments have not always been consistent on the weight to be attached to P’s past and present wishes and feelings, there is an emerging jurisprudence which accords them a special place in best interests decision-making.

Parallel concerns with the place of P’s wishes and values are expressed by John Coggon in his article, ‘Mental Capacity Law, Autonomy, and Best Interests: An Argument for Conceptual and Practical Clarity in the Court of Protection’. Examining recent judgments, Coggon is more critical of whether ‘best interests’ jurisprudence can be said to have achieved the ‘patient centred’ approach to best interests that he suggests was envisaged in the Act and which was endorsed by Baroness Hale in *James*. Arguing that the binary construction of autonomy, whereby the right to decide is contingent upon ‘capacity,’ should be rejected, Coggon presents an argument that P’s values (if known) ought to carry equal weight, whether the patient is regarded as having or lacking decision-making capacity. Parity between patients judged to have capacity and those assessed as not having capacity would certainly bring the MCA jurisprudence closer to the values of the UNCRPD. Like Donnelly, Coggon finds positive examples of patient-centred approaches, suggesting an evolution in MCA jurisprudence and a departure from medicalised constructions of best interests.

In 2007, Parliament introduced the Deprivation of Liberty Safeguards (DOLS), a new legal framework designed to close the so-called ‘Bournewood’ gap.^15^ Tagged onto the MCA through the Mental Health Act 2007, the DOLS have proved to be a controversial innovation. Indeed, it is perhaps in this respect that the MCA’s recent legacy has been most problematic. The mechanics of the DOLS are labyrinthine and they have a complex relationship with the Mental Health Act 1983. Since the Supreme Court’s decision in *Cheshire West and Cheshire Council v P*,^16^ there has been an explosion in number of applications for DOLS ‘standard authorisations’ in England, adding further strain to an already-creaking system.^17^ Since they first came into effect in 2009, calls to reform the DOLS have grown in volume. Later this year, the Law Commission will publish its recommendations for a new framework of safeguards which may redraw the boundary between the MCA and mental health law in England.^18^ In his paper, John Fanning analyses the DOLS and argues that the ‘continuities of risk’ which characterise them seem incongruous when set against the backdrop of the MCA and, more recently, the CRPD. Fanning claims that considerations of the risks that compliant yet incapacitated patients may pose to themselves or others, determine the nature and intensity of their interaction with mental health services. These considerations, he says, are implicitly relevant for the purposes of the DOLS’ best interests and eligibility requirements and retain a degree of paternalism in the decision-making process. Fanning argues that the DOLS in this way sit on a continuum of mental health laws whose operation depends on assessments of risk. Consequently, the interface between the DOLS and the Mental Health Act operates at times according to an ‘escalator’ dynamic; the bigger the risks, the more coercive the intervention. This colonisation by risk of decision-making processes outside the realm of the Mental Health Act is problematic and Fanning argues that even the Law Commission’s recent reform proposals would do little to bring a post-DOLS framework into line with the non-discriminatory ethos of the CRPD. Fanning concludes that more must be done to reconcile English mental health law with the principles of capacity, autonomy, and non-discrimination.

The breadth of the MCA’s application was always going to pose formidable challenges in terms of managing compliance. The lack of enforcement mechanisms and organisations with overall responsibility for oversight of the MCA in practice was a primary criticism levied by the House of Lords Select Committee. With hindsight, it might also be said that the MCA’s decision-making framework has not quite been the panacea that was originally envisaged. Minors under 16 years are still subject to the ‘*Gillick* test’, the refusal of life saving treatment for those who are 16 or 17 years old is still dealt with at common law,^19^ vulnerable adults, even though not lacking decision-making capacity, are apparently subject to the surviving inherent jurisdiction of the High Court^20^ and other groups are also potentially subject to tests other than those set out in the MCA.^21^ Recent years have seen an emerging jurisprudence from the CoP which champions the right to decide in cases where P is assessed as having capacity, such as *Kings College Hospital NHS Foundation Trust v C & Anor,*^22^ where Justice MacDonald went to great lengths to examine P’s world view and upheld her right to make the ‘unwise’ decision to refuse life sustaining dialysis after a failed suicide attempt at the age of 49. Losing the will to live because life had lost its ‘sparkle’, was not an indicator of incapacity. As the authors in this special issue concur, increasingly CoP judgments are recognising these rights in cases where the presumption of capacity has been rebutted. As Peter Jackson J in the case of *Wye Valley NHS Trust v B^23^* made clear, a finding that P lacks capacity should not be treated as an ‘off switch’ with regards to his rights and freedoms. The same sentiments are to be found in an earlier judgment by Hedley J in *An NHS Trust v P* where he stated that the intention behind the MCA was ‘not to dress an incapacitous person in forensic cotton wool but to allow them as far as possible to make the same mistakes that all other human beings are at liberty to make and not infrequently do’.^24^ It is only to be hoped that the Law Commission’s proposal that the MCA be amended so as to give greater priority to P’s wishes and feelings in best interests decision-making will be realised.^25^

As it enters its difficult teenage years, the MCA faces new challenges, not least the continuing instalments in the *Cheshire West* story, including the CoP’s efforts to introduce streamlined procedures for non-contentious deprivation of liberty applications.^26^ But whatever its imperfections, and whatever difficulties are faced by those who must apply it, the significant advances of the MCA and the still evolving CoP jurisprudence should not be lost on us.

## Footnotes

1 Select Committee on the Mental Capacity Act 2005, ‘Mental Capacity Act 2005: Post-Legislative Scrutiny’ (2014) HL Paper 139 <http://www.publications.parliament.uk/pa/ld201314/ldselect/ldmentalcap/139/139.pdf> accessed 11 August 2016.

2 Mental Capacity Act 2005, s 1(1).v

3 ibid, ss 1(5) and 4.

4 ibid, s 4.

5 [2013] UKSC 67, particularly Baroness Hale [45].

6 House of Lords Select Committee (n 1) para 104.

7 For example, ‘Agony of Woman Locked Up by Secret Court for Trying to Get Dad Out of Care Home’ *Daily Mail* (24 April 2013) and ‘That Sinister Court Mocks Justice Again’ *The Telegraph* (31 January 2015).

8 Namely Sir James Munby’s Practice Note of January 2014—*Practice Guidance (Transparency in the Court Of Protection)* [2014] EWCOP B2, particularly para 16: ‘Permission to publish a judgment should always be given whenever the judge concludes that publication would be in the public interest and whether or not a request has been made by a party or the media.’

9 *V v Associated Newspapers Ltd* [2016] EWCOP 21.

10 *GN v Newland* [2015] EWCOP 43 [1].

11 What Williams refers to as ‘the concertina effect’: V Williams and others, *Making Best Interest Decisions: People and Processes* (Mental Health Organisation 2012) 3.2. These safeguards include: s 1(2) the presumption of capacity; the s 1(3) duty to take ‘all practicable steps’ to enable people to make decisions for themselves (one of the most crucial provisions in delivering the ethos of full participation in decision-making, although rarely referred to in judgments); and s 1(4) preserving the right to make ‘unwise decisions’.

12 House of Lords Select Committee (n 1) para 53.

13 See art 12(4) of the UNCRPD in particular, and for discussion, see L Series, ‘Relationships, Autonomy and Legal Capacity’ (2015) 40 Intl J L & Psychiatry 80 and T Jütten, *Does the MCA Respect the Will and Preferences of the Disabled Person?* (Essex Autonomy Project, 2014) <http://autonomy.essex.ac.uk/uncrpd> accessed 11 August 2016.

14 cf W Martin and others, *Achieving CRPD Compliance: Is the Mental Capacity Act of England and Wales Compatible with the UN Convention on The Rights of Persons with Disabilities? If Not, What Next?* (Essex Autonomy Project, 2014) <http://autonomy.essex.ac.uk/uncrpd> accessed 11 August 2016.

15 *R v Bournewood Community and Mental Health NHS Trust, ex parte L* [1999] 1 AC 458; *HL v United Kingdom* (2005) 40 EHRR 32.

16 [2014] UKSC 19.

17 See Health & Social Care Information Centre, *Mental Capacity Act (2005): Deprivation of Liberty Safeguards (England) Annual Report 2014-15* (29 September 2015) <www.hscic.gov.uk/catalogue/PUB18577/dols-eng-1415-rep.pdf> accessed 23 June 2016.

18 See eg Law Commission, ‘Mental Capacity and Deprivation of Liberty: A Consultation Paper*’* (2015) Consultation Paper No 222; Law Commission, ‘Mental Capacity and Deprivation of Liberty: Interim Statement’ (25 May 2016) <www.lawcom.gov.uk/wp-content/uploads/2016/06/mental_capacity_interim_statement.pdf> accessed 22 June 2016.

19 *An NHS Foundation Trust v P* [2014] EWHC 1650.

20 Confirmed 8 years after the MCA was passed in *A Local Authority v DL* [2012] EWCA Civ 253.

21 E Cave, ‘Mind the Gaps: Determining Mental Capacity to Make Medical Treatment Decisions’ (2015) 36(1) Statute L Rev 86.

22 [2015] EWCOP 80.

23 [2015] EWCOP 60.

24 [2013] EWHC 50 (Fam) [10] and cited with approval by the Court of Appeal in *IM v LM* [2014] EWCA Civ 37.

25 See n 17, 1.41.

26 *Re X* [2014] EWCOP 25 (although disapproved in *Re X* [2015] EWCA Civ 599) and *Re NRA & Others* [2015] EWCOP 59.

